# Hepatic Cryotherapy for Cancer: A Reviewand Critique

**DOI:** 10.1155/1996/52565

**Published:** 1996

**Authors:** D. L. Morris

**Affiliations:** UNSW Department of Surgery The St. George Hospital Kogarah Sydney NSW 2217 Australia

## Abstract

*Background:* Wedge or other nonanatomic hepatic resections, performed in an attempt to spare functional parenchyma, often are not accomplished with clear resection margins and may be complicated by hemorrhage from the depth of the resection.

*Study Design:* The current study describes a technique of cryoassisted hepatic resection that allows for controlled resection with well-defined margins. The early experience in managing 16 tumors in 13 patients is reported.

*Results:* A cryoprobe is inserted into the tumor and freezing performed to a predetermined resection margin using ultrasound control. The ice ball, so formed, is then maintained and excised. The management of these 13 patients was associated with one intraoperative and two postoperative complications, including a death of a patient with cirrhosis who had infected ascites and died as a result of hepatic failure.

*Conclusions:* Cryoassisted hepatic resection seems to be safe and allows resection with good tumor clearance and maximal preservation of functional parenchyma.


### HEPATIC CRYOTHERAPY FOR CANCER: A REVIEW AND CRITIQUE

### ABSTRACT

Polk, W., Fong, Y., Karpeh, M. and Blumgart, L.H. (1995) A technique for the use of cyrosurgery to assist hepatic resection. Journal of the American College of Surgeons; 180: 171–176

***BACKGROUND*: Wedge or other nonanatomic hepatic resections, performed in an attempt to spare functional parenchyma, often are not accomplished with clear resection margins and may be complicated by hemorrhage from the depth of the resection.**
***STUDY DESIGN*: The current study describes a technique of cryoassisted hepatic resection that allows for controlled resection with well-defined margins. The early experience in managing 16 tumors in 13 patients is reported.**
***RESULTS*: A cryoprobe is inserted into the tumor and freezing performed to a predetermined resection margin using ultrasound control. The ice ball, so formed, is then maintained and excised. The management of these 13 patients was associated with one intraoperative and two postoperative complications, including a death of a patient with cirrhosis who had infected ascites and died as a result of hepatic failure.**
***CONCLUSIONS*: Cryoassisted hepatic resection seems to be safe and allows resection with good tumor clearance and maximal preservation of functional parenchyma.**
***J. Am. Coll. Surg.*, 1995, 180: 171–176.**

KEYWORDS: Hepatic resection cryosurgery hepatic colorectal metastases primary hepatocellular carcinoma.

### PAPER DISCUSSION

The use of cold to destroy cancers was described about 150 years[1] and was well understood 20 years ago[2] but advances in ultrasound[3] and cryomachine/probe technology have, however, allowed this to be applied to some deep cancers, including prostate, kidney and the topic of this essay, the liver. Before considering the paper of Polk *et al.* in detail, perhaps we could briefly review what is known of hepatic cryotherapy, and our own experience.

### INDICATIONS

### *1) Hepatoma*

There is a sizeable experience of cryotherapy for hepatoma in China[4] with approximately 20% five-year survival and no operative mortality. These patients did, however, receive other treatments, making the data difficult to interpret. The principal advantage of cryotherapy in this patient group is the preservation of hepatic parenchyma.

### *2) Liver Metastases From Colorectal Cancer*

This indication has been responsible for the majority of patients treated in the westen world[5]. My own experience is of 162 separate procedures in 149 patients, almost exclusively in unresectable patients, the few exceptions being mainly patients considered high risk for resection because of cardiovascular disease. Our technique is described elsewhere[6]. We have seen one thirty-day death (myocardial infarction) and one late death due to hepatic abscess associated with concurrent colectomy at 10 weeks.

Limited perioperative morbidity and short hospital stay was seen even in our early experience[7]. We have not had the devastating cryoshock phenomenon described by the Pittsburgh group[8] but we do not do a double freeze with complete thaw. There is no doubt that a twin freeze thaw has a considerably more destructive cellular effect[9,10] but even our partial twin freeze thaw produces greater evidence of hepatocellular injury and dramatic fall in platelet count[11,12]. We have chosen to only re-freeze the outer 1 cm of a cryolesion, which is the least definitively destroyed. The issue of the utility of regional chemotherapy following cryotherapy is covered elsewhere[13] but as this therapy is now proven to improve survival[14,15] and is reviewed elsewhere[16], we believe that, in patients with more than three lessions, it is mandatory.

CEA reduction has been seen in almost all secretors (82%) and the size of reduction is of median 84% of the preoperative value, the percentage reduction has been highly prognostic of survival[13]. In more than one half of the CEA responders, this was to the normal range and median survival in this group is in excess of 1000 days, which has to be regarded as most encouraging in patients with multiple (median 3.9) unresectable hepatic metastases[17].

The Boston group have mainly treated patients with operable lesions. Of 34 patients treated by cryotherapy alone, there were 1–3 lesions (mean 1.4)[5,18] and, of the first 49 patients treated in Boston, 35 were considered to have no gross residual disease after cryotherapy. Of these, 13 have died, although three deaths were from non-malignant casuses and, of the remaining 22, only 36% appear tumor free at 3–88 months (median 12). I do not yet believe that this follow-up is adequate for cryotherapy to be advocated in patients with operable lesions who have no medical risk factors for resection. I also believe it is premature to mount controlled trails to address this issue. Liver resection can achieve five-year survival in almost a half of our patients and with a mortality of well under 5%, so what does cryotherapy have to offer to this group? Even when five-year survival of larger numbers of patients with operable disease treated by cryotherapy is known, will cryotherapy offer any significant reduction in risk of the procedure: in skilled hands, I doubt it.

*3) Neuroendocrine*

Although a rare tumour, the role of cryotherapy for neuroendocrine liver metastases is perhaps most exciting and has involved some extensive and time-consuming surgery. We have to date treated without mortality six patients with unresectable hepatic metastases from neuroendocrine tumor. Not only have we seen excellent clinical, radiologic and tumor marker results[19] but the length of benefit in most of this group of patients seems to be considerable.

*4) Adjunct to liver resection: edge freeze/ contralateral disease*

We have used cryotherapy in a different way than the featured paper to treat involved or inadequate hepatic resection margins, where disease free survival is only 20% of the former group at 1 year[20], and to treat contralateral disease following lobectomy[21] with to date an apparently lower risk of edge recurrence than might be expected. Only two of twelve edge freeze patients have recurrence at a median of 14 months and, whilst the data is less mature, at least some of the contralobe freeze patients remain tumor free.

*5) Future*

Hepatic cryotherapy has already been done under laparoscopic guidance[22] but, although we have failed to produce gas embolism in a rabbit model[23], I remain concerned about the efficacy and safety of this (it really bleeds, sometimes) and also the ease of placement of an arterial perfusion device.

Cryotherapy does offer the ability to destroy large volumes of liver with relative safety. Our data suggests that the adequacy of tumor ablation, as measured by CEA reduction, does affect survival. Indeed, the large group where CEA returns to normal appear (at least as far as three years) to have similar survival prospects to patients who do have resectable disease. Cryotherapy has been a very useful adjunct to liver resection in our hands, although we have used it in a very different manner than Blumgart's group.

### CRITIQUE

The use of cryotherapy to ensure an adequate resection margin is hard for me to understand. Cryotherapy does take time, the equipment is expensive and it does have intra-operative hazards of its own, even if some of the postoperative sequelae of cryotherapy are avoided by this iceballectomy. Clearly, the concept depends upon the ultrasonographic skill of the operator because one will be making a judgement of the 1 cm margin between lesion edge and iceball edge on ultrasound and, of course, lesion edge disappears as soon as it is enveloped by iceball. It must be understood that this 1 cm margin needs to be on every margin of the tumor. The paper is also largely devoid of meaningful data, in that there is no effort to describe disease free recurrence. It is particularly disappointing that there is not even a measure of the actual resection margins achieved. i.e., minimum margin in each lesion. Perhaps it would be valuable if compared with some retrospective data for wedges. The contemporary condemnation of wedges in favour of segmentally based resection is also based upon the poor blood loss record of wedges. It seems unlikely that the described technique will alter this and there is not a comprehensive description of the blood loss/transfusion requirements of these patients in this paper.

The cryoablative role of hepatic cryotherapy may well require more data to convince us but its role as a handle or a fender bar would seem less likely to be of importance, but I've been wrong before.

### REFERENCES

1. Arnott, J. (1851) On the treatment of cancer by the regulated application of an anaesthetic temperature. London; J. Churchill, pp 32–54.
2. Cooper, I. S. (1963) Cryogenic surgery. A new method of destruction or extirpation of benign or malignant tissues. *New England Journal of Medicine,* **268**, 743–749.
3. Onik, G., Kane, R., Steele, G., McDermott, W. *et al.* (1986) Monitoring hepatic cryosurgery or malignant tissues. *Australian Journal of Radiology,* **147**, 665–669.
4. Zhou, X. D., Tang Z. Y., Yu, Y. Q., Ma, Z. C. (1988) Clinical evaluation of cryosurgery in the treatment of primary liver cancer. Reports of 60 cases. *Cancer,* **61**, 1889–1892.
5. Steele, G. (1994) Cryoablation in hepatic surgery. *Seminars in Liver Disease,* **14**, 120–125.
6. Ross, W. B., Horton, M., Bertolino, P., Morris, D. L. (1995) Cryotherapy of liver tumours – a practical guide. *HPB Surgery,* **8** (3), 167–173.
7. Goodie, D. B., Horton, M. D. A., Morris, R., Nagy, L. S., Morris, D. L. (1992) The anaesthetic experience with hepatic cryotherapy for metastatic colonic carcinoma. *Anaesthesia and Intensive Care,* **20**(4), 491–496.
8. Onik, G., Rubinsky, B., Zemel, R., Weaver, L., Diamond, D., Cobb, C., Porterfield, B. (1991) Ultrasound guided hepatic cryosurgery in the treatment of metastatic colon carcinoma. Preliminary results. *Cancer,* **67**, 901–907.
9. Gill, W., Frazer, J., Carter D. C. (1968) Repeated freeze thaw cycles in cryosurgery. *Nature,* **219**, 410–4.
10. Dilley, A. V, Dy, D. Y., Walters, A., Copeland, S., Gillies, A.E., Morris, R. W., Gibb, D. B., Cook, T. A., Morris, D. L. (1993) Laboratory and animal model evaluation of the Cryotech LCS 2000 in hepatic cryotherapy. *Cryobiology,* **30**, 74–85.
11. Stewart, G. J., Preketes, A. P., Horton, M. D., Ross, W. B., Morris, D. L. (1995) Hepatic cryotherapy: double freeze cycles achieve greater hepatocellular injury in man. *Cryobiology,* **32**, 215–9.
12. Cozzi, P. J., Stewart, G. J., Morris, D. L. (1994) Thrombocytopenia after hepatic cryotherapy for colorectal metastases correlates with hepatocellular injury. *World Journal of Surgery,* **18** (5), 774–777.
13. Preketes, A. P., King, J., Caplehom, J. R. M., Clingan, P. R., Ross, W. B., Morris, D. L. (1994) CEA reduction after cryotherapy for liver metastases from colon cancer predicts survival. *Australian and New Zealand Journal of Surgery,* **64**, 612–614.
14. Rougier, P., Laplanche, A., Huguier, M. (1992) Hepatic arterial infusion of floxuaridine in patients with liver metastases from colorectal carcinoma. Long term results of a prospective randomised trial. *Journal of Clinical Oncology,* **10**, 1112–1118.
15. Allen-Mersh, T. G., Earlam, S., Fordy, C., Abrams, K., Houghton, J. (1994) Quality of life and survival with continuous hepatic artery floxuridine infusion for colorectal liver metastases. *Lancet,* **344**, 1255–1260.
16. McCall, J. and Morris, D.L., Hepatic Artery Chemotherapy for Colorectal Liver Metastases. *Aust NZ J Surg* 1995; **65**(6): 383–389. ISSN 0004 86–82.
17. Morris, D.L., Ross, W. B. Hepatic cryotherapy for primary and secondary liver cancer. Oncology Clinics of North America, In Press.
18. Kane, R. A. (1993) Ultrasound-guided hepatic cryosurgery for tumor ablation. *Seminars in International Radiology,* **10**, 133–142.
19. Cozzi, P.J., Englund, R., Morris, D. L. (1995) Cryotherapy treatment of patients with hepatic metastases from neuroendocrine tumours. *Cancer,* **76**, 501–9.
20. Cady, B., Stone, M. D., McDermott, V., Jenkins, R. C., Boethe, A., Lavin. P. T., Lovett, E. J., Steele, G. D., Technical and biological factors in disease free survival after hepatic resection for colorectal cancer metastases. *Archives of Surgery* **127**, 561–569.
21. McCall, J. L., Ross, W. B., Morris, D. L. (1994) Cryotherapy with liver resection for the treatment of liver tumours. Gut 1994, 35(Suppl 2): S15.
22. Cushieri, A. (1995) Laparoscopic management of cancer patients. Journal of the Royal College of Surgeons of Edinburgh, **40**, 1–9.
23. McCall, J. L., Jorgensen, J. O., Morris, D. L. (1994) Laparoscopic hepatic cryotherapy: a preliminary safety study in rabbits. *Australian and New Zealand Journal Surgery,* **65**, 429.

D. L. Morris
UNSW Department of Surgery
The St. George Hospital
Kogarah, Sydney NSW 2217
Australia

